# Very Small Embryonic-Like Stem Cells, Endothelial Progenitor Cells, and Different Monocyte Subsets Are Effectively Mobilized in Acute Lymphoblastic Leukemia Patients after G-CSF Treatment

**DOI:** 10.1155/2018/1943980

**Published:** 2018-06-21

**Authors:** Andrzej Eljaszewicz, Lukasz Bolkun, Kamil Grubczak, Malgorzata Rusak, Tomasz Wasiluk, Milena Dabrowska, Piotr Radziwon, Wojciech Marlicz, Karol Kamiński, Janusz Kloczko, Marcin Moniuszko

**Affiliations:** ^1^Department of Regenerative Medicine and Immune Regulation, Medical University of Bialystok, Ul. Waszyngtona 13, 15-269 Bialystok, Poland; ^2^Department of Haematology, Medical University of Bialystok, Ul. Waszyngtona 13, 15-276 Bialystok, Poland; ^3^Department of Hematological Diagnostics, Medical University of Bialystok, Ul. Waszyngtona 13, 15-276 Bialystok, Poland; ^4^Regional Centre for Transfusion Medicine, Ul. M. Skłodowskiej-Curie 23, 15-950 Bialystok, Poland; ^5^Department of Gastroenterology, Pomeranian Medical University, Ul. Unii Lubelskiej 1, 71-252 Szczecin, Poland; ^6^Department of Cardiology, Medical University of Bialystok, Ul. Waszyngtona 13, 15-276 Bialystok, Poland; ^7^Department of Population Medicine and Prevention of Civilization Diseases, Medical University of Bialystok, Ul. Waszyngtona 13, 15-276 Bialystok, Poland; ^8^Department of Allergology and Internal Medicine, Medical University of Bialystok, Ul. Waszyngtona 13, 15-276 Bialystok, Poland

## Abstract

**Background:**

Acute lymphoblastic leukemia (ALL) is a malignant disease of lymphoid progenitor cells. ALL chemotherapy is associated with numerous side effects including neutropenia that is routinely prevented by the administration of growth factors such as granulocyte colony-stimulating factor (G-CSF). To date, the effects of G-CSF treatment on the level of mobilization of different stem and progenitor cells in ALL patients subjected to clinically effective chemotherapy have not been fully elucidated. Therefore, in this study we aimed to assess the effect of administration of G-CSF to ALL patients on mobilization of other than hematopoietic stem cell (HSCs) subsets, namely, very small embryonic-like stem cells (VSELs), endothelial progenitor cells (EPCs), and different monocyte subsets.

**Methods:**

We used multicolor flow cytometry to quantitate numbers of CD34+ cells, hematopoietic stem cells (HSCs), VSELs, EPCs, and different monocyte subsets in the peripheral blood of ALL patients and normal age-matched blood donors.

**Results:**

We showed that ALL patients following chemotherapy, when compared to healthy donors, presented with significantly lower numbers of CD34+ cells, HSCs, VSELs, and CD14+ monocytes, but not EPCs. Moreover, we found that G-CSF administration induced effective mobilization of all the abovementioned progenitor and stem cell subsets with high regenerative and proangiogenic potential.

**Conclusion:**

These findings contribute to better understanding the beneficial clinical effect of G-CSF administration in ALL patients following successful chemotherapy.

## 1. Background

Acute lymphoblastic leukemia (ALL) is a malignant disease of lymphoid progenitor cells, characterized by accumulation of lymphoblasts in the bone marrow. Standard therapeutic procedure involves use of chemotherapy to first induce remission and next reduce tumor burden and kill residual cells in the bone marrow [1]. Chemotherapy is associated with numerous side effects including neutropenia, and therefore granulocyte colony-stimulating factor (G-CSF) is routinely used in order to improve neutrophil renewal [2]. However, chemotherapy causes considerable damage to many other cells, such as different progenitor and stem cells and tissues. Activation of regenerative processes requires involvement of stem and progenitor cells that could initiate mechanisms improving cell renewal, development of new vasculature, and tissue reconstruction. However, chemotherapy used in ALL depletes not only stem cells in the bone marrow but also stem cells in the vascular niche of the bone marrow [3, 4]. The bone marrow-associated vascular niche plays a key role in supporting such hematopoiesis processes as hematopoietic stem cell (HSC) maintenance, differentiation, and transendothelial migration. Multiple signaling and adhesion molecules are involved in vascular niche homeostasis, including Jag-1, Notch, c-kit, SCF, angiopoietin-1 (Ang-1), and Tie-2. Importantly, recent reports indicated a role of Ang-1/Tie2 signaling in vascular niche regulation and regeneration [5].

The bone marrow is a reservoir of numerous stem and progenitor cells, both hematopoietic and nonhematopoietic (non-HSCs). Adult HSCs were the first identified and thoroughly characterized group of stem cells in humans [6]. To date, HSCs are the ones of few stem cell subtypes that are used routinely in clinical practice worldwide [7]. On the other side, non-HSCs are comprised of several different populations of stem and progenitor cells including endothelial progenitor cells (EPCs) and very small embryonic-like stem cells (VSELs) [8–10]. It has been hypothesized that adult EPCs are delivered from HSCs, while VSELs represent distinct population of adult pluripotent stem cells [9, 11]. Although bone marrow-delivered VSELs do not exhibit direct hematopoietic activity, they can acquire hematopoietic potential under specific conditions, and thereby they may support bone marrow renewal [12]. Similarly, release of VSELs to the circulation can contribute to supporting regenerative processes in distal tissues. In contrast to VSELs, EPCs are primarily involved in supporting vascularization processes [13, 14]. The role of EPCs in the promotion of angiogenesis and revascularization is usually supported by other cell types including pericytes and proangiogenic subsets of monocytes. In addition, proangiogenic monocytes, similarly to EPCs, were shown to support local stem and progenitor cell differentiation in the bone marrow [15, 16].

Granulocyte colony-stimulating factor (G-CSF) was the first cytokine identified and introduced into medical practice in order to treat neutropenia and to induce mobilization of hematopoietic stem cells (HSC) in donors before transplantation. Physiological plasma levels of G-CSF are either very low or undetectable; however, G-CSF can be produced locally by many tissues in response to proinflammatory signaling mediated by IL-1b, TNF, and IFN-*β*, among others [2, 17]. Moreover, locally produced G-CSF affects neutrophil function by increasing their survival in inflamed/infected tissue by apoptosis inhibition. Furthermore, release of G-CSF into the circulation stimulates neutrophil production and their release from the bone marrow [18]. On the other hand, the recent report of our group indicated that repetitive G-CSF administration to pediatric patients effectively induced significant mobilization of EPCs and putative proangiogenic monocytes [19]. Similarly, other reports showed that G-CSF may be used to improve outcome of patients with cardiovascular events [20]. However, to date, the effect of G-CSF treatment on VSELs and EPCs mobilization in immunocompromised patients has not been evaluated. Therefore, we hypothesized that the use of G-CSF in the prevention of chemotherapy-induced neutropenia in ALL patients may support regeneration of damaged tissues by mobilization of stem and progenitor cells. In this report, we aimed to assess the effect of G-CSF administration on the mobilization of VSELs, EPCs, and proangiogenic monocyte subsets in ALL patients with complete remission after chemotherapy. Furthermore, we set out to analyze the effects of G-CSF administration on chemotactic factors for VSELs, EPCs, and proangiogenic monocytes, namely, SDF-1 and angiopoietins.

## 2. Methods

### 2.1. Patients

21 patients with diagnosed acute lymphoblastic leukemia B linage and 12 age-matched normal donors were enrolled in the study. Patients' median age at the time of sample collection was 39 (21–58). Diagnoses were established according to the 2008 WHO recommendation [21]. Blood counts, flow cytometry, molecular study, FISH, and cytogenetic analysis were performed, reviewed, and classified. Patients were treated at the Department of Haematology, Medical University of Bialystok from 2013 to 2016, with induction and 2 consolidation chemotherapy regimens corresponding to the standard therapy based on the Polish Adult Leukemia Group [22]. All included patients were in complete remission and had no minimal residual disease after the induction < 0.1% and consolidation < 0.01% [22]. After induction, the response was evaluated in accordance with the recommendation by NCCN Guidelines. G-CSF (Neupogen) was given s.c. at the dose of 5 *μ*g/kg, for 7 days, as a prophylaxis of neutropenia. The samples were collected before stimulation and at the 8th day following treatment. All samples were collected upon the approval of the Ethics Committee of the Medical University of Bialystok.

### 2.2. Flow Cytometry

Freshly obtained EDTA-anticoagulated whole blood specimens were stained by using panel of mouse anti-human monoclonal antibodies (Table 1), according to stain-and-then-lyse-and-wash protocol as previously described [19, 23]. Briefly, 100 *μ*L (for monocytes) and 175 *μ*L (for EPC and VSELs) of whole blood were stained with monoclonal antibodies (Table 1) and incubated for 30 min at room temperature, in the dark. Thereafter, 2 mL of FACS lysing solution (Becton Dickinson Bioscience) was added for erythrocyte lysis, followed by 15 min incubation in the dark. Next, cells were washed twice with cold PBS (phosphate-buffered saline, Corning) and fixed with CellFix (BD Biosciences). For all stainings, appropriate fluorescence-minus-one (FMO) controls were used for setting compensation and to assure correct gating. Samples were analyzed with FACSCalibur flow cytometer (BD Biosciences). Obtained data were analyzed using FlowJo ver. 7.6.5 software (Tree Star) as previously described [19, 23].

### 2.3. Cytokine Assay

SDF-1, angiopoietin-,1 and angiopoietin-2 plasma levels were quantified by means of commercially available enzyme-linked immunosorbent assays (ELISA, DuoSet, R&D) in 96-well plates. Samples were directly assayed according to manufacturer's instructions. Protein levels in the specimens were calculated from a reference curve generated using appropriate protein standards. Finally, the plates were analyzed with automated light absorbance reader (LEDETEC 96 system). The results were calculated by MicroWin 2000 software.

### 2.4. Statistics

Statistical analysis was performed by using GraphPad Prism 6 (GraphPad software). Mann–Whitney *U* test was used to compare differences among groups, while Wilcoxon test was used to compare changes in course of G-CSF treatment. Additionally, Spearman correlation coefficient was used to determine correlations between plasma protein levels and analyzed cell subsets. The differences were considered statistically significant at *p* < 0.05. The results are presented as medians and interquartile range.

## 3. Results

**Figure 1:**
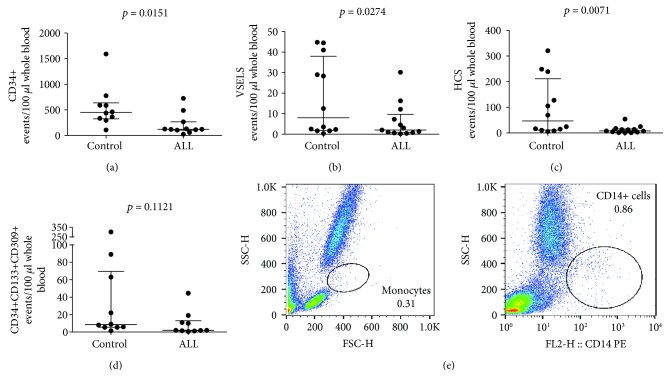
Summary of flow cytometry analyses of (a) CD34+ progenitor cells (CD34+ cells), (b) very small embryonic-like stem cells (VSELs, lin-CD235a-CD45-CD133+), (c) hematopoietic stem cells (HSC, VSELs, lin-CD235a-CD45+CD133+), and (d) endothelial progenitor cell (EPCs, CD34+CD133+CD309+ cells) numbers in normal donors (control) and ALL patients after successful chemotherapy before G-CSF treatment; Mann–Whitney *U* test was used; (e) representative flow cytometry dot plots of ALL patients after successful chemotherapy before G-CSF treatment.

First, we analyzed the numbers of all progenitor and stem cells identified as CD34+ cells as well as HSCs, VSELs, EPCs, and frequencies of monocyte subsets in ALL patients after chemotherapy before G-CSF administration, and we compared these values with age-matched control subjects. We found decreased numbers of CD34+ progenitor cells (Figure 1(a)), VSELs (Figure 1(b)), and HSCs (Figure 1(c)) in ALL patients as compared to healthy donors. Interestingly, we found no differences in the numbers of EPCs delineated by CD34+CD133+CD309+ phenotype (Figure 1(d)) between ALL patients and healthy subjects. Importantly, CD14+ cells could not be detected in the peripheral blood of 19 ALL patients after chemotherapy (Figure 1(e)).

**Figure 2:**
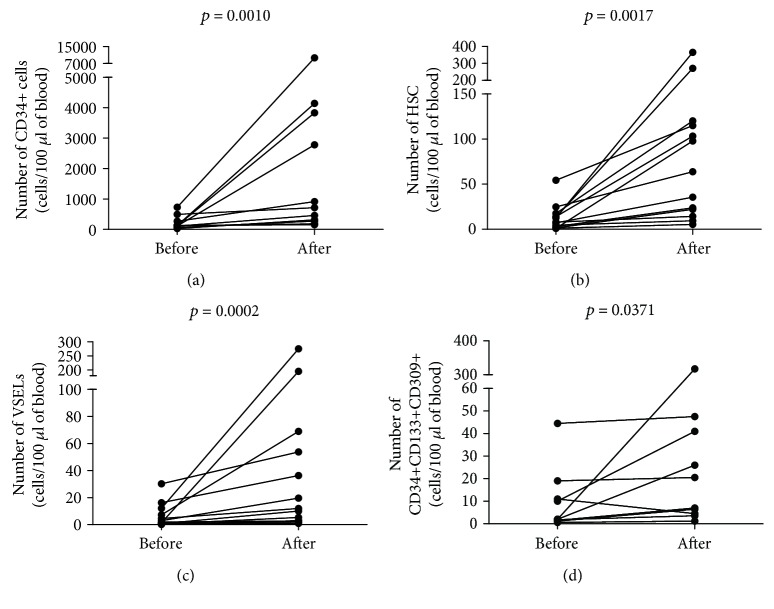
Changes in (a) CD34+ progenitor cells (CD34+ cells), (b) very small embryonic-like stem cells (VSELs, lin-CD235a-CD45-CD133+), (c) hematopoietic stem cells (HSC, lin-CD235a-CD45+CD133+), and (d) endothelial progenitor cell (EPCs, CD34+CD133+CD309+ cells) numbers in ALL patients after successful chemotherapy before and after G-CSF treatment; Wilcoxon test was used.

**Figure 3:**
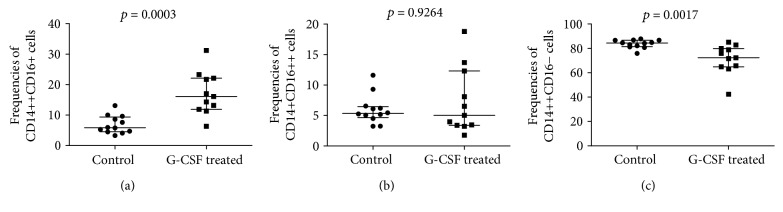
Summary of flow cytometry analyses of (a) intermediate (CD14++CD16+), (b) nonclassical (CD14+CD16++), and (c) classical (CD14++CD16−) monocyte frequencies in normal donors (control) and ALL patients after successful chemotherapy and G-CSF treatment; Mann–Whitney *U* test was used.

Having found decreased numbers of analyzed stem and progenitor cells in immunocompromised patients, we next aimed to investigate effects of G-CSF treatment on hematopoietic stem cell mobilization in ALL patients. It should be noted that mobilization of hematopoietic/progenitor cells after G-CSF administration is usually delayed, with peak levels observed within 5–7 days [19]. As expected, we found significant increase in the numbers of CD34+ precursors and HSCs (from 122.5 (105.3–725.8) to 713.8 (270.9–3829), Figure 2(a), and from 7.843 (2.22–15.67) to 63.73 (18.21–117.5), Figure 2(b), resp.) in all analyzed individuals 7 days after initial treatment. Next, we evaluated the numbers of VSELs and EPCs (determined by linage-CD235a-CD45-CD133+ and CD34+CD133+CD309+ phenotype, resp.). Interestingly, we observed substantial increase of VSEL numbers after G-CSF administration from 2 (0.683–9.706) to 11.96 (2.85–61.37) (Figure 2(c)). Similarly, the numbers of EPCs increased from 2 (1.419–13) to 13.75 (4.257–42.63) (Figure 2(d)). Moreover, we observed substantial increase in CD14+ cell numbers after G-CSF treatment in all individuals (data not shown). Therefore, we next analyzed the composition of monocyte subsets after G-CSF therapy. We observed significantly higher frequencies of putative proangiogenic intermediate monocytes (delineated by CD14++CD16+ phenotype, Figure 3(a)), but not nonclassical CD14+CD16++ monocytes (Figure 3(b)) in G-CSF-treated ALL patients as compared to normal donors (16.10 (11.90–22.10) versus 5.86 (4.54–9.35)). Consequently, frequencies of classical monocytes were lower in ALL patients following G-CSF therapy in comparison to healthy subjects (72.30 (64.90–79.90) versus 84.35 (81.43–86.68), Figure 3(c)).

**Figure 4:**
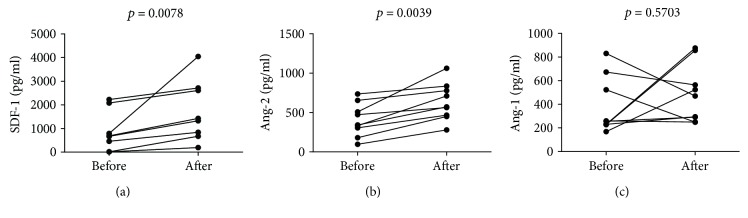
Changes in (a) SDF-1, (b) angiopoietin 2, and (c) angiopoietin 1 levels in ALL patients after successful chemotherapy before and after G-CSF treatment; Wilcoxon test was used.

In parallel, we evaluated levels of SDF-1 and two major angiopoietins, namely, Ang-1 and Ang-2. We observed that G-CSF administration increased SDF-1 (Figure 4(a)) and Ang-2 (Figure 4(b)), but not Ang-1 (Figure 4(c)) plasma levels in all analyzed individuals. Finally, we investigated whether plasma SGF-1, Ang-1, and Ang-2 levels were correlated to numbers of CD34+ cells, HSC, VSELs, EPCs, and different monocyte subsets. Interestingly, we found that CD34+ cell numbers correlated positively with Ang-1 and SDF-1 levels (*p* = 0.0372, *r* = 0.5110, and *p* = 0.0383, *r* = 0.5545, resp.). Surprisingly, HSC and VSEL numbers correlated positively only with Ang-1 (*p* = 0.0257, *r* = 0.5297) and Ang-2 (*p* = 0.0208, *r* = 0.5944) plasma levels, respectively. Furthermore, we found positive correlation between absolute numbers of CD14++CD16+, but not CD14+CD16+ monocytes and SDF-1 levels (*p* = 0.0187, *r* = 0.1000).

## 4. Discussion

Here, we demonstrated that chemotherapy regimens that are routinely used in ALL patients decreased the numbers of circulating CD34+ cells and, more specifically, HSCs, and VSELs, but did not affect EPC numbers. Thus, we found that the administration of G-CSF to immunocompromised adult patients is capable of inducing mobilization of progenitor and stem cells with high regenerative potential into the periphery. Moreover, we showed that G-CSF administration caused significant increases in plasma levels of SDF-1 and Ang-2, but not Ang-1.

Within the bone marrow, all progenitor cells reside in separated microenvironmental niches, which control their proliferation, differentiation, and release to the circulation [24]. High-dose chemotherapy used in ALL treatment directly induces regression of the bone marrow and destroys its ability to produce and release blood cells, namely, leukocytes, red blood cells, and platelets, as well as different subsets of progenitor cells involved in regeneration process, including EPCs and VSELs. This is also the main cause of neutropenia [25]. It is well established that progenitor cell recovery is highly dependent on the number of chemotherapy cycles. Interestingly, peak levels of these cells after chemotherapy correlated with the rate and extent of platelet recovery [26]. On the other hand, it is believed that regeneration of the bone marrow vascular niche is crucial for proper reconstruction of hematopoiesis after chemotherapy. Notably, bone marrow endothelial cells were shown to support differentiation of hematopoietic progenitors and their mobilization to the periphery [27, 28]. Therefore, EPCs may be one of the first subsets involved in the regeneration of the bone marrow, and their release into the circulation may improve vascularization of distal tissues damaged by chemotherapy. Similarly, decreased numbers of VSELs in the peripheral blood of immunocompromised patients may be a consequence of their contribution to the restoration of hematopoiesis in the bone marrow. More importantly, as presented in this study, the process may be further supported by G-CSF application.

Notably, acquisition of hematopoietic function by VSELs was reflected by changes in the expression of certain genes that regulate hematopoietic processes, including PU-1, c-myb, LMO2, and Ikaros. In a consequence, VSELs differentiated into CD45+ hematopoietic cells. However, normal bone marrow-delivered VSELs express numerous markers characteristic for pluripotent cells, including SSEA-1, Rexo-1, Rif-1, Nanog, and Oct-4, and can be differentiated *in vitro* into cells of all three germ layers, namely, ectoderm, endoderm, and mesoderm [9, 29]. Importantly, VSELs are mobilized from the bone marrow into the circulation in response to tissue injury, including myocardial infraction and ischemia [30, 31]. Therefore, they are believed to support regeneration process in many degenerative conditions. Similarly to our results, in experimental mouse model, the administration of exogenous G-CSF resulted in increased mobilization and release of VSELs from the bone marrow. This might be a result of SDF-1 signaling, since VSELs were shown to express CXCR4 [32]. Surprisingly, in this study we found no significant correlation between the number of VSELs and SDF-1 plasma levels. Interestingly, previous observations in inflammatory bowel disease showed that VSELs and EPCs mobilization can occur in SDF-1-independent manner [33]. Furthermore, we showed that increased Ang-2 levels are, somehow, related to increased VSEL mobilization. However, further studies are needed to explain this phenomenon.

It was previously reported that EPC mobilization from the bone marrow to the periphery depends on SDF-1 signaling [34]. Interestingly, however, we found no association between the SDF-1 levels and numbers of EPCs. Furthermore, all endothelial cells were shown to express angiopoietin receptor (Tie-2) that may be activated by both angiopoietin-1 (Ang-1) and angiopoietin-2 (Ang-2). Kopp and collaborators showed that Ang-1 stimulated Tie-2 expression in the bone marrow vasculature, and thus they may play a role in the promotion of hematopoiesis. Of the four already described angiopoietins, Ang-1 and Ang-2 are to date the best characterized ones [35]. Ang-1 was recognized as principal activator of Tie-2, while Ang-2 acted as Tie-2 inhibitor that causes destabilization of blood vessels, what constituted the initial stage of vascularization process [36]. Notably, Tie-2 is expressed not only on EPCs but also on HSCs and small subset of CD16+ monocytes, namely, Tie-2-expressing monocytes (TEMs). In fact, intermediate CD14++CD16+ monocytes represent the predominant population of peripheral blood Tie-2-expressing cells [37]. Furthermore, reparative monocytes with proangiogenic potential were found to express SDF-1 receptor, namely, CXCR4 [38]. Importantly, Capoccia and collaborators showed that G-CSF-mobilized monocytes were able to induce vascularization at sites of ischemia [17]. In some contrast to our results, Murdoch and collaborators indicated that Ang-2 may serve as chemotactic factor for monocytes with proangiogenic potential [39]. Further studies supported these findings showing that Ang-2 signaling markedly enhanced proangiogenic activity of TEMs [40]. Thus, we hypothesized that G-CSF treatment in ALL patients after successful chemotherapy can increase numbers of reparative and proangiogenic monocytes in SDF-1-dependent manner; however, their activity may be controlled by increased Ang-2 signaling. However, further studies are still warranted in order to assess this mechanisms in more detail.

## 5. Conclusions

In summary, we showed that G-CSF treatment in immunocompromised patients induced efficient mobilization of stem and progenitor cells with high regenerative and proangiogenic potential. These findings could help to better understand beneficial clinical effects of G-CSF therapy in immunocompromised patients. Our findings suggest that G-CSF treatment can be considered as additional tool used in patients after chemotherapy in order to support recovery process. However, further studies are still needed to assess safety of such therapeutic approach in different clinical settings.

## Figures and Tables

**Table 1:** Characteristic of used monoclonal antibodies.

Marker	Fluorochrome	Host	Clone	Manufacturer
CD14	PE	Mouse anti-human	MφP9	Becton Dickinson Bioscience
CD16	FITC	Mouse anti-human	B73.1	Becton Dickinson Bioscience
CD34	FITC	Mouse anti-human	581	Becton Dickinson Bioscience
CD45	PE	Mouse anti-human	HI30	Becton Dickinson Bioscience
CD133	APC	Mouse anti-human	AC133	Miltenyi Biotec
CD235a	FITC	Mouse anti-human	GA-R2 (HIR2)	Becton Dickinson Bioscience
CD309	PE	Mouse anti-human	89,106	Becton Dickinson Bioscience

PE: phycoerythrin; FITC: fluorescein isothiocyanate; APC: allophycocyanin.

## Data Availability

The datasets used and/or analyzed during the current study are available from the corresponding author on reasonable request.
